# Rifampicin resistance and mortality in patients hospitalised with HIV-associated tuberculosis

**DOI:** 10.4102/sajhivmed.v23i1.1396

**Published:** 2022-09-27

**Authors:** Ruan Spies, Charlotte Schutz, Amy Ward, Avuyonke Balfour, Muki Shey, Mark Nicol, Rosie Burton, Bianca Sossen, Robert Wilkinson, David Barr, Graeme Meintjes

**Affiliations:** 1 Department of Medicine, New Somerset Hospital, Cape Town, South Africa; 2 Department of Medicine, Faculty of Health Sciences, University of Cape Town, Cape Town, South Africa; 3 Wellcome Centre for Infectious Diseases Research in Africa (CIDRI-Africa) and Institute of Infectious Disease and Molecular Medicine, Faculty of Health Sciences, University of Cape Town, Cape Town, South Africa; 4 Division of Infection and Immunity, School of Biomedical Sciences, University of Western Australia, Perth, Australia; 5 Médecins sans Frontières, Cape Town, South Africa; 6 The Francis Crick Institute, London, United Kingdom; 7 Department of Infectious Disease, University College London, London, United Kingdom; 8 Institute of Infection and Global Health, University of Liverpool, Liverpool, United Kingdom

**Keywords:** HIV-associated tuberculosis, rifampicin-resistant tuberculosis, drug-resistant tuberculosis, multi-drug resistant TB, TB, Khayelitsha Hospital

## Abstract

**Background:**

Patients with HIV and drug-resistant tuberculosis (TB) are at high risk of death.

**Objectives:**

We investigated the association between rifampicin-resistant TB (RR-TB) and mortality in a cohort of patients who were admitted to hospital at the time of TB diagnosis.

**Method:**

Adults hospitalised at Khayelitsha Hospital and diagnosed with HIV-associated TB during admission, were enrolled between 2013 and 2016. Clinical, biochemical and microbiological data were prospectively collected and participants were followed up for 12 weeks.

**Results:**

Participants with microbiologically confirmed TB (*n* = 482) were enrolled a median of two days (interquartile range [IQR]: 1–3 days) following admission. Fifty-three participants (11.0%) had RR-TB. Participants with rifampicin-susceptible TB (RS-TB) received appropriate treatment a median of one day (IQR: 1–2 days) following enrolment compared to three days (IQR: 1–9 days) in participants with RR-TB. Eight participants with RS-TB (1.9%) and six participants with RR-TB (11.3%) died prior to the initiation of appropriate treatment. Mortality at 12 weeks was 87/429 (20.3%) in the RS-TB group and 21/53 (39.6%) in the RR-TB group. RR-TB was a significant predictor of 12-week mortality (hazard ratio: 1.88; 95% confidence interval: 1.07–3.29; *P* = 0.03).

**Conclusion:**

Mortality at 12 weeks in participants with RR-TB was high compared to participants with RS-TB. Delays in the initiation of appropriate treatment and poorer regimen efficacy are proposed as contributors to higher mortality in hospitalised patients with HIV and RR-TB.

## Introduction

Drug-resistant tuberculosis (DR-TB) is a global health concern.^1^ In South Africa (SA), 13 005 cases of rifampicin-resistant TB (RR-TB) were identified in 2019.^2,3^ People living with HIV are at higher risk of acquiring DR-TB, which is associated with poorer treatment outcomes in this patient population.^2,4,5,6^

The treatment of DR-TB has low success rates and high mortality due to lengthy, poorly tolerated and poorly efficacious regimens.^7,8^ In 2018, only 58% of patients with DR-TB globally were successfully treated.^1^ Mortality estimates from studies conducted primarily in outpatients or in TB hospitals in high-burden settings, prior to the introduction of bedaquiline-based regimens, range from 11% to 39%.^9,10,11,12,13,14^ The introduction of bedaquiline-based treatment has been associated with a reduction in mortality in patients with DR-TB; however, treatment failure and mortality remain high in high-prevalence settings, with mortality still ranging from 6% to 17%.^15,16,17,18,19,20,21^

As most DR-TB is managed in outpatient clinics or in TB hospitals, most research on DR-TB outcomes occurs in these settings. Patients who are hospitalised with HIV-associated TB are often severely ill and inpatient case fatality rates range from 11% to 32%.^22,23,24,25,26,27^ In autopsy series, inpatient deaths occur with a median of 4–5 days following admission.^28,29,30^ There are scarce data on outcomes of patients diagnosed with HIV-associated DR-TB admitted to general hospitals.

The outcomes of a cohort of patients admitted to hospital in Khayelitsha, SA, newly diagnosed with HIV-associated TB, were previously described.^31^ In this cohort, RR-TB was present in 16.9% of participants who died and in 7.2% of survivors.^31^ In this secondary analysis, we aimed to describe the association between RR-TB and mortality in patients diagnosed with HIV-associated TB while hospitalised and to identify factors associated with mortality in patients with RR-TB.

## Methods

### Study design and setting

Patients admitted to Khayelitsha Hospital were enrolled to a prospective cohort study between January 2013 and October 2016. The hospital is in Khayelitsha – a large township in Cape Town, SA, with high rates of HIV, TB and DR-TB.^32^ Most cases of TB are managed in primary healthcare clinics; however, patients may be referred to hospital if they require inpatient care.

### Participants

All patients in the emergency unit and medical wards were screened on weekdays. Adults with HIV infection, a CD4 count of < 350 cells/µL and a high clinical suspicion of TB were eligible to enrol into the study. Pregnant patients, patients who received anti-TB therapy within the past month, or patients who were recently initiated and received three or more doses of anti-TB therapy, were not eligible for enrolment. Clinical details, chest X-ray, sputum, urine and blood samples were obtained at enrolment. Additional samples, including extra-pulmonary samples such as pleural fluid, cerebrospinal fluid and lymph node aspirates, were obtained as indicated by clinical staff. Participants remained under routine clinical care and treatment decisions were made by clinical staff and not by study staff. Results of validated sputum, urine and blood TB tests that were collected by the study were communicated to the routine clinical team. Participants were assessed daily in the ward and, after discharge, were managed in primary care according to local guidelines. Participants had a telephonic follow-up at week 4 and returned for a clinical assessment at week 12.

### Laboratory assays

Tuberculosis microbiology was performed by the National Health Laboratory Services (NHLS). Sputum and urine were sent for TB culture and Xpert MTB/RIF assay (Cepheid, Sunnyvale, California, United States). The study preceded the introduction of Xpert MTB/RIF Ultra, which has a higher sensitivity than Xpert MTB/RIF Ultra for pulmonary TB.^33^ Mycobacterial blood culture was performed by culturing 5 mL of whole blood in Myco/F Lytic (Becton Dickinson Biosciences, Franklin Lakes, New Jersey, United States) bottles for 42 days. The GenoType MTBR*plus* assay (Hain Lifesciences, Nehren, Germany) was used to identify *Mycobacterium tuberculosis* complex from the positive sputum and blood cultures and provided rifampicin and isoniazid resistance testing. RR isolates underwent susceptibility testing to second-line drugs at a referral laboratory. Between 2014 and 2016, phenotypic resistance testing was performed for amikacin and ofloxacin. From 2016 the GenoType MTBDRsl (Hain Lifesciences, Nehren, Germany) line probe assay was used to assess susceptibility to aminoglycosides and fluoroquinolones. CD4 count, HIV viral load, haemoglobin, creatinine and electrolytes, liver function and C-reactive protein (CRP) tests were performed on all participants.

### Data collection and definitions

Clinical data were captured on standardised case record forms from patient interviews, hospital folders and clinical review at enrolment. The primary outcome was survival at 12 weeks. If patients could not be contacted at 12 weeks, searches of electronic records were conducted. Participants with an electronic entry indicating a clinic visit, collection of medicine or a laboratory test performed beyond 12 weeks were assumed to be alive at week 12. Participants without electronic entries at or beyond 12 weeks were classified as lost to follow-up. In this study we describe and analyse the subset of patients with microbiologically confirmed TB, defined as participants with *M. tuberculosis* identified by culture or Xpert MTB/RIF from any clinical sample. RR-TB was defined as rifampicin resistance on any sample using either of the genotypic tests performed at the NHLS while rifampicin-susceptible TB (RS-TB) was defined as rifampicin susceptibility on all samples.

### Statistical analyses

Data were analysed using R version 4.02 (R Core Team, Vienna, Austria).

Median values with interquartile ranges were used as measures of central tendency and dispersion. Categorical variables were described using counts and proportions and were compared using the Fisher’s exact test. Continuous variables were compared between study groups using the Mann-Whitney U test.

To investigate the association of RR-TB with 12-week mortality, we hypothesised that RR-TB could cause mortality through two mechanisms: (1) delayed initiation of effective therapy and (2) lower efficacy of DR-TB therapy in preventing early mortality. We therefore treated receipt of appropriate anti-TB therapy (i.e. rifampicin-based therapy for RS-TB, any DR-TB therapy for RR-TB and an individualised regimen for extremely drug-resistant [XDR] TB) as a time-dependent variable in a Cox proportional hazards model, where hazard of mortality after start of appropriate therapy was compared to hazard of mortality prior to initiation of appropriate therapy, capturing effect of delay to appropriate treatment initiation. This allowed assessment of the association between RR-TB and mortality after adjusting for an effect of RR-TB on delay to appropriate treatment initiation.

We further hypothesised that measures of disease severity at baseline (hypoxia defined as peripheral oxygen saturation < 94% on room air, serum creatinine, sodium, protein gap [PG] [defined as the difference between total protein and albumin], haemoglobin, CD4 count, HIV viral load, Glasgow coma scale [GCS] < 15 and weight) could confound TB treatment status and mortality (with patients presenting more unwell being initiated on anti-TB therapy more urgently). Finally, we considered that patient factors associated with both RR-TB and mortality, including age and sex, and the above markers of baseline disease severity could confound the relationship between RR-TB and mortality. Both these sets of variables were therefore included as covariates in the model.

Continuous predictor variables were log-transformed to resolve highly skewed distributions, and because proportional or multiplicative changes in the values of these variables were thought to be more biologically meaningful. Peripheral oxygen saturation was dichotomised to reflect a non-linear relationship between oxygen saturation and partial pressure of oxygen as parsimoniously as possible.

**FIGURE 1:**
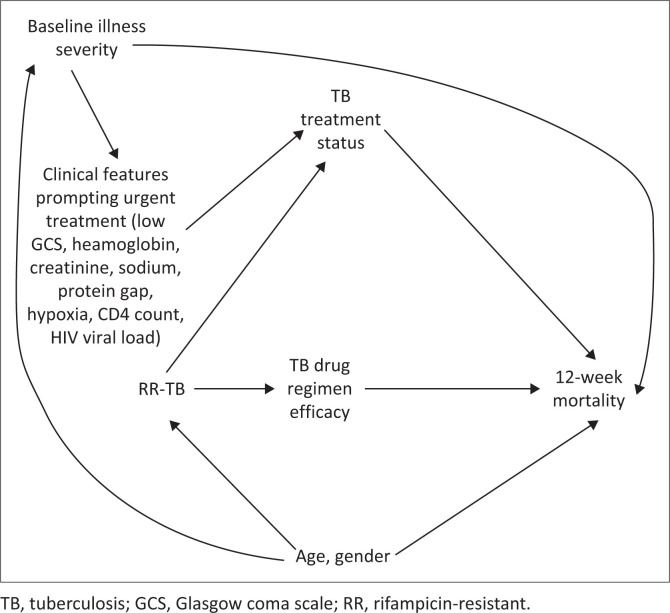
Hypothesised causal structure for the relationship between rifampicin-resistant tuberculosis and 12-week mortality.

The hypothesised causal structure is summarised in a directed acyclic graph (Figure 1).

Observations on variables included in Cox regression were all > 95% complete except for weight which was missing in 8% of participants. All missing values were considered missing completely at random or missing at random after adjusting for other observed covariates and single-imputed for model fitting using classification and regression trees implemented with the *mice* package in R.

Participants lost to follow-up were censored on their last day of contact with health services. Kaplan-Meier plots were used to estimate survival over the study period, stratified by rifampicin-sensitivity status.

### Ethical considerations

This study was approved by the University of Cape Town Human Research Ethics Committee (reference number 057/2013). Participants provided written informed consent where possible. The process involved in enrolling patients who did not have the capacity to provide informed consent has been described previously.

## Results

### Participant characteristics

A total of 482 participants with HIV-associated, microbiologically confirmed TB were included in this analysis, enrolled a median of two days (interquartile range [IQR]: 1–3 days) after admission to hospital. Fifty-three participants (11.0%) had RR-TB. Of these, 16 (30.0%) had TB resistant to rifampicin only and 32 (60.0%) had multi-drug resistant TB, three (6.0%) of whom had TB resistant to a second-line drug (one participant had XDR-TB and two participants had pre-XDR-TB). Five (9.0%) participants provided samples that were identified as RR by GeneXpert MTB/RIF but the results of further drug susceptibility testing were unavailable. In the RS-TB group, 194 (45.2%) tested positive for *M. tuberculosis* by Xpert MTB/RIF compared to 22 (41.5%) participants in the RR-TB group. Thirteen (2.7%) participants were lost to follow-up over the 12-week study period. Demographic and clinical characteristics, stratified by rifampicin susceptibility, are displayed in Table 1. Characteristics for the RR-TB group, stratified by vital status, are shown in Table 2.

**TABLE 1:** Demographic and clinical characteristics and outcomes, stratified by rifampicin susceptibility status.

Characteristic	Rifampicin-susceptible						Rifampicin-resistant						*P*
	*n*	Median	Mean	%	IQR	s.d.	*n*	Median	Mean	%	IQR	s.d.	
**Total**	429	-	-	-	-	-	53	-	-	-	-	-	
**Gender**													**0.030**
Female	220	-	-	51.0	-	-	19	-	-	36.0	-	-	-
Male	209	-	-	49.0	-	-	34	-	-	64.0	-	-	-
**Age**		36.0			31–43			37.0			32–44	-	0.600
**ART status**											-	-	0.090
Interrupted	95	-	-	22.0	-	-	16	-	-	30.0	-	-	-
Naïve	183	-	-	43.0	-	-	15	-	-	28.0	-	-	-
On ART	149	-	-	35.0	-	-	21	-	-	40.0	-	-	-
Unknown	2	-	-	0.5	-	-	1	-	-	1.8	-	-	-
**TB history**													
First TB	255	-	-	61.0	-	-	15	-	-	30.0	-	-	**< 0.001**
Previous TB	161	-	-	39.0	-	-	35	-	-	70.0	-	-	
**Weight (kg)**	-	54.0	-	-	48–63	-	-	49.0	-	-	43–55	-	**< 0.001**
**GCS < 15**	54	-	-	13.0	-	-	12	-	-	23.0	-	-	**0.050**
**CD4 Count (cells/μL)**	-	53.0	-	-	20–112	-		41.0	-	-	11–103	-	0.200
**HIV viral load (copies/mL) log**	-	5.3	-	-	3.9–5.8	-	-	4.9	-	-	3.0–5.6	-	**0.040**
**Total protein (g/L)**	-	75.0	-	-	67–83	-	-	71.0	-	-	65–79	-	0.060
**Albumin (g/L)**	-	24.0	-	-	21–28	-	-	24.0	-	-	20.2–27.0	-	0.440
**Creatinine ( μ mol/L)**	-	80.0	-	-	61–126	-	-	81.0	-	-	59–107		0.440
**Sodium (mmol/L)**	-	128.0	-	-	124–131	-	-	129.0	-	-	125–131		0.490
**Haemoglobin (g/dL)**	-	8.4	-	-	7–10	-	-	8.4	-	-	7.2–10.3		0.500
**CRP (mg/L)**	-	-	170	-		105–233			163			121–230	0.800
**Peripheral oxygen saturation < 94 %**	30	-	-	7.1	-	-	6	-	-	11.0	-	-	0.300
***Mycobacterium tuberculosis* cultured from blood**	190	-	-	45.0	-	-	25	-	-	47.0	-	-	0.800
**Urine LAM positive**	166	-	-	45.0	-	-	27	-	-	59.0	-	-	0.040
**Urine GeneXpert positive**	201	-	-	55.0	-	-	26	-	-	59.0	-	-	0.400
**Outcome at week 12**													0.010
Alive	336	-	-	78.0	-	-	30	-	-	57.0	-	-	-
Dead	87	-	-	20.0	-	-	21	-	-	40.0	-	-	-
Lost to follow-up	6	-	-	1.4	-	-	2	-	-	3.8	-	-	-
**Most likely cause of death**													0.200
Complications of TB	39	-	-	44.0	-	-	11	-	-	55.0	-	-	-
Other opportunistic infections	9	-	-	10.0	-	-	4	-	-	20.0	-	-	-
Other	18	-	-	20.0	-	-	1	-	-	5.0	-	-	-
Sepsis	23	-	-	26.0	-	-	4	-	-	20.0	-	--	-
**Immune reconstitution inflammatory syndrome**	1	-	-	0.2	-	-	1	-	-	1.9	-	-	-

Note: *P*-values in bold are statistically significant.

TB, tuberculosis; IQR, interquartile range; s.d., standard deviation; ART, antiretroviral treatment; GCS, Glasgow coma scale; CRP, C-reactive protein; LAM, lipoarabinomannan assay.

**TABLE 2:** Demographic and clinical characteristics of patients with rifampicin-resistant tuberculosis, stratified by vital status at 12 weeks.

Characteristic	Alive						Dead						*P*
	*n*	Median	Mean	%	IQR	s.d.	*n*	Median	Mean	%	IQR	s.d.	
**Total**	32	-	-	60.4	-	-	21	-	-	39.6	-	-	-
**Gender**													0.50
Female	11	-	-	34.0	-	-	9	-	-	43.0	-	-	-
Male	21	-	-	66.0	-	-	12	-	-	57.0	-	-	-
**Age**		37			31–42			37			32–44		0.70
**ART status**													0.06
Interrupted	11	-	-	34.0	-	-	5	-	-	24.0	-	-	-
Naïve	12	-	-	38.0	-	-	3	-	-	14.0	-	-	-
On art	9	-	-	28.0	-	-	12	-	-	57.0	-	-	-
Unknown	0	-	-	-	-	-	1	-	-	4.8	-	-	-
**TB history**													0.80
First TB	10	-	-	33.0	-	-	6	-	-	30.0	-	-	-
Previous TB	20	-	-	67.0	-	-	14	-	-	70.0	-	-	-
**Weight (kg)**	-	50	-	-	46–56	-	-	44	-	-	40–54	-	0.08
**GCS < 15**	6	-	-	20.0	-	-	6	-	-	29.0	-	-	0.50
**CD4 count (cells/μL)**	-	60			21–112	-	-	28	-	-	5–72	-	0.07
**HIV viral load (copies /mL) log**	-	4.9	-	-	3.0–5.7	-	-	4.05	-	-	2.5–5.4	-	0.58
**Total protein (g/L)**	-	70	-	-	65–76	-	-	74	-	-	66–79	-	0.50
**Albumin (g/L)**	-	24	-	-	21–27	-	-	22	-	-	20–26	-	0.37
**Creatinine ( μ mol/L)**	-	80	-	-	86–100	-	-	96	-	-	59–136		0.31
**Sodium (mmol/L)**	-	130	-	-	125–131	-	-	129	-	-	125–131		0.90
**Haemoglobin (g/dL)**	-	8.5	-	-	7.5–10.4	-	-	8	-	-	7.0–10.2	-	0.45
**CRP (mg/L)**	-	-	145	-	-	84–216	-	-	190	-	-	147–276	**0.05**
**Peripheral oxygen saturation < 94 %**	3	-	-	9.4	-	-	2	-	-	9.5	-	-	0.92
***Mycobacterium tuberculosis* cultured from blood**	11	-	-	34.0	-	-	13	-	-	62.0	-	-	**0.05**
**Urine LAM positive**	18	-	-	60.0	-	-	9	-	-	56.0	-	-	0.82
**Urine GeneXpert positive**	15	-	-	54.0	-	-	11	-	-	69.0	-	-	0.31

Note: *P*-values in bold are statistically significant.

TB, tuberculosis; IQR, interquartile range; s.d., standard deviation; ART, antiretroviral treatment; GCS, Glasgow coma scale; CRP, C-reactive protein; LAM, lipoarabinomannan assay.

### Time to treatment initiation and treatment status

**FIGURE 2:**
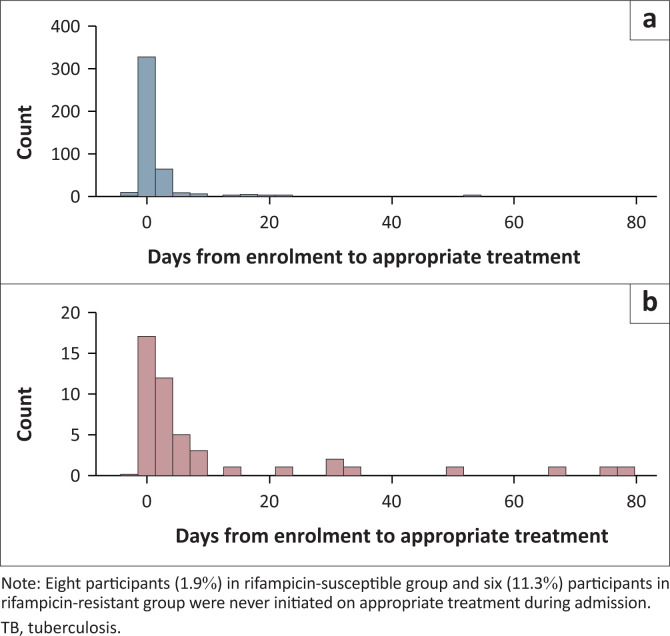
Histogram of time to appropriate treatment initiation stratified by rifampicin susceptibility status. (a) Rifampicin-susceptible TB, (b) Rifampicin-resistent TB.

Participants in the RS-TB group initiated appropriate treatment (rifampicin-based therapy for RS-TB, any DR-TB therapy for RR-TB and an individualised regimen for XDR-TB) a median of 1 day following enrolment (IQR: 1–2 days) compared to three days in the RR-TB group (IQR: 1–9 days), *P* < 0.001. Eight (1.9%) participants in the RS-TB group were not initiated on appropriate treatment during their admission, six of whom died. Six (11.3%) participants in the RR-TB group died prior to the initiation of appropriate treatment (Figure 2). Four of the six participants died within three days of enrolment. In the two remaining participants, RR-TB was confirmed on blood culture – the results of which were only available after their deaths.

### Mortality

**FIGURE 3:**
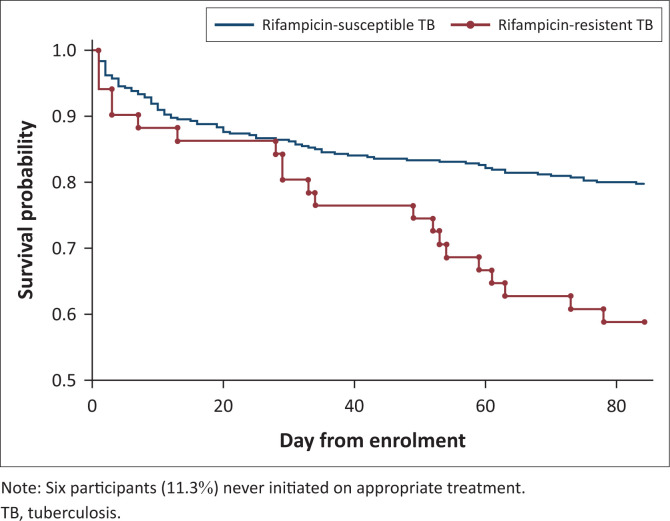
Kaplan-Meier estimate of survival function, stratified by rifampicin susceptibility status.

Mortality at 12 weeks was 87/429 (20.3%) in the RS-TB group and 21/53 (39.6%) in the RR-TB group (*P* = 0.008). The Kaplan-Meier curve in Figure 3 demonstrates the relationship between rifampicin susceptibility and time-to-death. Table 3 depicts the results of the Cox proportional hazards model which modelled the relationship between RR-TB and 12-week mortality. RR-TB was significantly associated with 12-week mortality (hazard ratio 1.88; 95% confidence interval 1.07–3.29; *P* = 0.03) when adjusting for CRP, CD4 count, HIV viral load, creatinine, sodium, PG, haemoglobin, weight, GCS, hypoxia, age, sex, and TB treatment status as a time-dependent variable to adjust for treatment delay.

**TABLE 3:** Cox proportional hazards model of association between rifampicin-resistant tuberculosis and 12-week mortality, adjusted for markers of illness severity, tuberculosis treatment status and anticipated confounders.

Variable	Hazard ratio	95% confidence interval	*P*
On appropriate TB treatment	0.90	0.40–2.01	0.800
CD4 count (cells/μL) log	0.88	0.75–1.03	0.100
HIV viral load (copies/mL) log	0.99	0.93–1.04	0.600
GCS < 15	1.51	0.92–2.48	0.100
Serum creatinine (μmol/L) log	**1.87**	**1.43–2.45**	**< 0.001**
Serum sodium (mmol/L) log	0.98	0.94–1.02	0.400
Hypoxia†	1.59	0.78–3.23	0.200
Haemoglobin (g/dL) log	**0.43**	**0.19–0.98**	**0.040**
Protein gap log‡	1.00	0.98–1.02	0.800
Male gender	0.70	0.46–1.06	0.090
Age (years) log	**3.75**	**1.67–8.41**	**< 0.010**
Weight (kg) log	0.48	0.18–1.29	0.150
**Rifampicin - resistant TB**	**1.88**	**1.07–3.29**	**0.030**

Note: Log defined as logarithm with base 10. Bold values denote statistically significant association variables.

TB, tuberculosis; GCS, Glasgow coma scale.

† , Defined as peripheral oxygen saturation < 94%;

‡ , Calculated as serum total protein (g/L).

serum albumin (g/L)

## Discussion

In this study we describe 482 patients with HIV-associated, microbiologically confirmed TB, diagnosed while admitted to a general hospital. We found a high prevalence of RR-TB in the study cohort and a high 12-week mortality in participants with RR-TB. Rifampicin-resistant TB was significantly associated with mortality, even when controlling for markers of disease severity and delay to treatment initiation.

The mortality rate we describe is significantly higher than described in other studies of patients with HIV-associated RR-TB. Unlike our study, these studies included participants both with and without HIV, longer follow-up periods and participants in predominantly outpatient or TB hospital settings. Prospective cohorts of outpatients with RR-TB, with follow-up periods ranging 6–24 months, describe mortality rates of 9% – 31%, with HIV prevalence 38% – 85%.^34,35,36,37^ Retrospective cohorts of patients with RR-TB in TB hospitals describe mortality rates of 18% – 29%, with HIV prevalence 52% – 100%.^38,39,40^ Retrospective studies of outpatients describe mortality rates of 13% – 21%, with HIV prevalence 70% – 100%.^10,12,13^

In our cohort, 29% of participants with RR-TB who died did so prior to the initiation of a DR-TB regimen. This suggests that a substantial proportion of acutely ill patients with DR-TB die as inpatients, before a diagnosis of DR-TB can be confirmed, before DR-TB treatment regimens can be initiated and before they are registered on the national TB programme. These patients would contribute to the recognised gap between numbers of DR-TB diagnoses and numbers registered in the DR-TB programme.^3^ The authors hypothesised that the high mortality rates they observed could be driven by two, not necessarily mutually exclusive, mechanisms: delays to initiation of appropriate treatment for RR-TB and less effective therapies for RR-TB compared to RS-TB.

In this study, time to initiation of appropriate treatment was shorter than described in previous studies,^9,41^ likely reflecting access to rapid diagnostics, multiple different samples taken for TB testing, and regular monitoring available in hospital. Although the difference in time to treatment initiation between the RS-TB and RR-TB groups was statistically significant, the difference is of unknown clinical significance. Several factors may have contributed to delays in appropriate treatment initiation in the RR-TB group including delays in specimen collection (patients in the RR-TB group may have been sicker at baseline and thus sputum sample acquisition for microbiological testing may have been more difficult), in awaiting the results of TB culture and drug susceptibility testing and in initiating appropriate treatment once these became available. Previous studies have failed to demonstrate a reduction in mortality in RR-TB with a reduction in time to treatment initiation.^9,42^

In this study analysis, the effect of being on appropriate TB therapy on mortality during the follow-up period was unclear. This implies that the association between RR-TB and 12-week mortality observed in this cohort was not mediated by diagnostic delay. Despite the marginal difference in time to treatment initiation, the 12-week mortality in the RR-TB group was much higher. When adjusting for TB treatment status as a time-dependent variable, RR-TB remained significantly associated with 12-week mortality, suggesting that additional factors related to RR-TB, other than time to treatment initiation, were important. These additional factors could include residual confounding, but the finding is also consistent with lower efficacy of DR-TB regimens during the study period. Interestingly, the Kaplan-Meier survival curves for participants with RR-TB versus RS-TB (Figure 3) diverge at day 21 of follow-up, suggesting early factors, including delays to treatment initiation, are less important contributors to differences in mortality than later factors, such as poorer efficacy of DR-TB treatment regimens. This study preceded the introduction of bedaquiline-based regimens for patients with RR-TB and participants in our study received RR-TB regimens containing injectable second-line drugs which are poorly tolerated and which have poor efficacy.^8^ The introduction of bedaquiline-based regimens has resulted in promising improvements in survival in DR-TB;^17^ however, prospective data in hospitalised patients at high risk of early mortality are lacking. This study suggests that efficacy of evolving DR-TB regimens in seriously ill patients with HIV-associated TB is an important topic for future research.

This study has several limitations. Although our cohort was relatively large, the population of participants with RR-TB was small. The authors did not objectively measure adherence to TB treatment once participants had been discharged, potentially introducing uncertainty into their measurement of the association between treatment status and mortality. Due to the observational design of this study, the authors were unable to eliminate residual confounding when measuring the association between RR-TB and mortality and they cannot therefore make conclusions as to the causal nature of this relationship. The strengths of this study include its extensive TB testing and prospective follow-up, with vital status at 12 weeks identified for 98% of participants. This study also describes a unique, under-studied population of hospitalised patients with HIV-associated RR-TB and CD4 count < 350 cell/µL.

## Conclusion

This study describes a high 12-week mortality in patients admitted to hospital at the time of TB diagnosis with HIV-associated RR-TB. We suggest that delays in treatment initiation and poorly efficacious treatment regimens, prior to the introduction of bedaquiline as standard of care, are possible contributors to mortality in this population. Hospitalised patients with DR-TB represent an under-studied group with a high risk of early mortality. Research into the contributors to mortality in this population and improved diagnostic and therapeutic strategies are required.
